# High-Resolution 3D versus Standard-Resolution 2D T2-Weighted Turbo Spin Echo MRI for the Assessment of Lumbar Nerve Root Compromise

**DOI:** 10.3390/tomography8010020

**Published:** 2022-01-24

**Authors:** Elisabeth Sartoretti, Thomas Sartoretti, Árpád Schwenk, Alex Alfieri, David Czell, Michael Wyss, Lukas Wildi, Christoph A. Binkert, Sabine Sartoretti-Schefer

**Affiliations:** 1 Institute of Radiology, Kantonsspital Winterthur, Brauerstrasse 15, 8401 Winterthur, Switzerland; elisabeth.sartoretti@uzh.ch (E.S.); arpad.schwenk@ksw.ch (Á.S.); christoph.binkert@ksw.ch (C.A.B.); sabine.sartoretti@ksw.ch (S.S.-S.); 2 Faculty of Medicine, University of Zürich, 8006 Zürich, Switzerland; david.czell@hin.ch; 3 Department of Radiology and Nuclear Medicine, Maastricht University Medical Center, Maastricht University, 6211 LK Maastricht, The Netherlands; 4 Institute of Neurosurgery, Kantonsspital Winterthur, 8401 Winterthur, Switzerland; Alex.alfieri@ksw.ch; 5 Philips Health Systems, 8810 Zürich, Switzerland; michael.wyss@ksw.ch; 6 Institute of Rheumatology, Kantonsspital Winterthur, 8401 Winterthur, Switzerland; lukas.wildi@ksw.ch

**Keywords:** spine, radiculopathy, magnetic resonance imaging, spinal nerve roots, acceleration

## Abstract

Radiculopathy can be caused by nerve root irritation and nerve root compression at the level of the lateral recess or at the level of the intervertebral foramen. T2-weighted (T2w) MRI is considered essential to evaluate the nerve root and its course, starting at the lateral recess through the intervertebral foramen to the extraforaminal space. With the introduction of novel MRI acceleration techniques such as compressed SENSE, standard-resolution 2D T2w turbo spin echo (TSE) sequences with a slice-thickness of 3–4 mm can be replaced with high-resolution isotropic 3D T2w TSE sequences with sub-millimeter resolution without prolonging scan time. With high-resolution 3D MRI, the course of the nerve root can be visualized more precisely due to a detailed depiction of the anatomical situation and less partial volume effects, potentially allowing for a better detection of nerve root compromise. In this intra-individual comparison study, 55 patients with symptomatic unilateral singular nerve root radiculopathy underwent MRI with both 2D standard- and 3D high-resolution T2w TSE MRI sequences. Two readers graded the degree of lumbar lateral recess stenosis and lumbar foraminal stenosis twice on both image sets using previously validated grading systems in an effort to quantify the inter-readout and inter-sequence agreement of scores. Inter-readout agreement was high for both grading systems and for 2D and 3D imaging (Kappa = 0.823–0.945). Inter-sequence agreement was moderate for both lumbar lateral recess stenosis (Kappa = 0.55–0.577) and lumbar foraminal stenosis (Kappa = 0.543–0.572). The percentage of high degree stenosis with nerve root deformity increased from 16.4%/9.8% to 41.8–43.6%/34.1% from 2D to 3D images for lateral recess stenosis/foraminal stenosis, respectively. Therefore, we show that while inter-readout agreement of grading systems is high for both standard- and high-resolution imaging, the latter outperforms standard-resolution imaging for the visualization of lumbar nerve root compromise.

## 1. Introduction

Lumbar nerve root radiculopathy is a common condition in clinical practice. Radiculopathy can be caused by nerve root irritation and nerve root compression at the level of the lateral recess or at the level of the intervertebral foramen [1]. In a healthy person, the nerve root passes through the lateral recess and enters the intervertebral foramen where the nerve root is surrounded by perineural fat, thus protecting the nerve root from compression of the adjacent osseous, ligamentous, and discal structures. In patients with narrowing of the lateral recess or narrowing of the intervertebral foramen, the space surrounding the nerve root is diminished either by bulging/herniation of the adjacent disc, hypertrophy of the adjacent flavum ligament, or by hypertrophy and osteophyte formation of the adjacent facet joint. Consequently, nerve root contact, displacement, deformity, and compression may develop, depending on the severity of the lateral recess or intervertebral foramen narrowing. Often, these processes lead to the typical clinical symptomatology of radiculopathy [2,3,4].

In patients with radiculopathy, MRI is considered the imaging modality of choice to visualize and evaluate the relationship between the nerve root and the lumbar foramen [5,6,7] and between the nerve root and the lateral recess [8].

Specifically, sagittal and transversal T2w images are considered essential to evaluate the nerve root and its course from the dural sac to the extraforaminal space. Traditionally, 2-dimensional (2D) T2w turbo spin echo (TSE) sequences with a slice thickness of 3–4 mm are acquired. However, with recent advances in MRI technology such as compressed sensing [9,10,11,12], spine MRI protocols can considerably be improved either in spatial resolution or in a reduction in scan time. For example, sagittal 2D sequences with 3–4 mm slice thicknesses can be replaced with high-resolution 3-dimensional (3D) thin slice sequences with isotropic, sub-millimeter resolution without prolonging the scan time.

High-resolution, submillimeter 3D acquisitions provide images with less partial volume effects compared to the original thick-sliced 2D sagittal acquisitions. Thus, the relationship between the nerve root and the surrounding structures within the lateral recess and within the foramen can potentially be identified more precisely [7].

After image acquisition, grading systems are then often employed to semi-quantitively describe the degree of pathology. In particular, the grading systems of Lee et al. [5] for lumbar foraminal stenosis and Pfirrmann et al. [8] for nerve root compromise at the level of the lateral recess in patients with disc herniation should be mentioned.

Recently, a study has introduced a new practical grading system for lumbar foraminal stenosis as seen in high-resolution submillimeter T2w images [7]. Therein, the authors state that the original grading system by Lee et al. is no longer suitable for the evaluation of high-resolution images as a far more complex relationship between the nerve root and the surrounding structures within the intervertebral foramen can be identified. However, the authors did not assess the reproducibility and applicability of their upgraded grading system for standard 2D images.

Furthermore, as this grading system allows for a comprehensive description of even the smallest changes in the lumbar foramen, a more reliable and accurate description of nerve root compromise may be achieved. Combined with high-resolution 3D imaging, an improved diagnosis of lumbar foraminal stenosis may potentially be achieved.

Thus, the aim of this study was two-fold: first, we sought to assess the applicability and reproducibility of this upgraded grading system for standard 2D T2w TSE imaging. Second, we sought to evaluate the inter-sequence agreement of grading scores between standard 2D and high-resolution 3D images in patients with symptomatic unilateral singular nerve root radiculopathy. Therein, we hypothesized that high-resolution imaging may enable a more reliable visualization of high-grade stenosis and nerve root compromise.

## 2. Materials and Methods

### 2.1. Study Subjects

Patients referred for MRI of the lumbar spine due to symptomatic unilateral singular nerve root radiculopathy were enrolled at two centers between May and September 2021. Only patients with unilateral single radiculopathy were enrolled in order to being able to concentrate on the compression of an individual nerve root and in order to being able to validate the imaging findings against the clinical symptomatology. Importantly, both potential nerve root compromise due to disc bulging, disc herniation, or osteo-disco-ligamentous compression in the lateral recess as well as in the foramen were evaluated. Patients with osteodiscoligamentous lumbar spinal canal stenosis were excluded.

Prior to MRI examination, all patients underwent a comprehensive clinical examination by a spine surgeon, neurologist, or rheumatologist (all board-certified in their specialty) that included a complete anamnesis and physical examination of the patient. Electromyography and preoperative block were performed in 23 cases to confirm the presence of radiculopathy. Sixteen patients underwent surgery following the complete diagnostic workup including physical/clinical examination and MRI.

Initially, 70 patients were considered for potential inclusion. Twelve patients were excluded due to osteodiscoligamentous lumbar spinal canal stenosis. Three patients were excluded because not all MRI sequences were acquired. Thus, 55 patients (29 men, 26 women, mean age of 57.3 years and range 25 to 89 years) were finally enrolled (44 from institution 1 and 11 from institution 2). Thirty patients presented with left sided and 25 patients with right sided radiculopathy. Neurological examination revealed an L1 radiculopathy in one patient, an L3 radiculopathy in six patients, an L4 radiculopathy in 10 patients, an L5 radiculopathy in 24 patients, and a S1 radiculopathy in 14 patients.

### 2.2. MRI Protocol

All patients underwent routine lumbar MRI examinations at one of three 1.5 T scanners (Achieva and Ingenia at center 1 and Ingenia at center 2, all from Philips) at two centers (Kantonsspital Winterthur, Switzerland and WIN4, Winterthur, Switzerland). Patients were examined in the supine position with slightly flexed knees. MRI protocols were identical at both centers and on all three MRI scanners.

The MRI protocol included a 2D T1w TSE sequence, the standard-resolution 2D T2w TSE sequence, the high-resolution 3D T2w TSE sequence (Spine View), and a STIR T2w TSE sequence. For the high-resolution 3D T2w TSE sequence, secondary curved transverse reconstructions with a slice thickness and increment of 1 mm were generated. Sequence parameters were selected based on the vendor’s recommendation after in-house and external optimization efforts.

Sequence parameters of the T2w sequences were as follows:

(a) 2D T2w TSE sequence: acquisition sagittal, repetition time (TR) 3000 ms, echo time (TE) 100 ms, flip angle 90°, echo train length 20, number of echoes 1, FOV 160 × 270 × 65 mm, slice thickness 4 mm, acquired voxel size 0.75 × 0.95 × 4.0 mm^3^, reconstructed voxel size: 0.63 × 0.63 × 4.0 mm^3^, number of signal averages (NSA) 2.0. Acquisition time 03 min 02 s.
(b) 3D T2w TSE sequence: acquisition sagittal, DRIVE pulse yes, TR 1300 ms, TE 95 ms, flip angle 90°, echo train length 50, number of echoes 1, field of view (FOV) 200 × 300 × 90 mm^3^, acquired voxel size 0.8 × 0.8 × 1.0 mm^3^, reconstructed voxel size 0.4 × 0.4 × 0.5 mm^3^, number of slices 180, acceleration: Compressed SENSE factor 7.0, NSA 1.0, acquisition time 04 min 46 s. This sequence was accelerated with the compressed SENSE acceleration technique that uses a variable density Poisson disk-sampling scheme followed by iterative reconstruction. A more detailed description of this acceleration technique can be found elsewhere [10,13]. The sequence was generally acquired in the sagittal plane of the section parallel to the spinal column. In patients with scoliosis, however, a secondary curved sagittal reconstruction parallel to the scoliotic lumbar vertebrae was obtained. Curved transverse reconstructions were computed parallel to the individual disc levels.

### 2.3. Image Evaluation

Images were evaluated by two readers (S.S.-S., board-certified neuroradiologist with 30 years of experience and TS, trainee with four years of experience) in consensus in a blinded and randomized manner. The readout was thereby performed twice with four weeks separating both sessions.

Readers evaluated the set of images one at a time with 2D and 3D T2w TSE images being evaluated separately and individually. Specifically, in each image set, readers were provided with one of the sagittal T2w TSE sequences as well as with axial curved reconstructions of the 3D T2w TSE sequence and with sagittal 2D T1w TSE images if required. Axial curved reconstructions of the 3D sequence were provided for both the 2D and 3D images sets as an axial 2D T2w sequence could not be acquired separately due to time constraints.

The symptomatic nerve root was marked on images with a small orange arrow prior to the readout. The symptomatic nerve root was identified based on clinical information provided by the referring physician and based on the radiological report drafted during clinical routine.

The presence of disc bulging, disc herniation, and osteo-disco-ligamentous narrowing were evaluated relative to the level of the radiculopathy [1]. A compression of a specific nerve root could either be caused by a nerve root compromise within the lateral recess at a level above the radiculopathy or by a nerve root compression within the intervertebral foramen at the level of the radiculopathy. This means that an L5 radiculopathy could be due to a compression of this nerve root at the L4–L5 level within the lateral recess or at the L5–S1 level within the intervertebral foramen. If a compression of the S1 nerve root was present, only compression of this nerve root within the lateral recess was evaluated.

The relationship between the nerve root and surrounding intervertebral foramen was classified by using the updated practical grading system for lumbar foraminal stenosis [7]. In brief, this is a 6-point grading system that is based on the widely used Lee classification [5]. Grade A indicates absence of foraminal stenosis; Grades B, C, D, and E indicate the presence of foraminal stenosis with contact of the nerve root with surrounding anatomical structures (on one, two, three or four sides for B, C, D, and E, respectively), but without morphological change in the nerve root. To each grade, a number code indicating the location of contact between the nerve root and surrounding anatomical structure(s) is appended: 1, 2, 3, and 4 indicate contact of the nerve root at the superior, posterior, inferior, and anterior position of the borders of the lumbar foramen, respectively. Grade F indicates the presence of foraminal stenosis with morphological change and compression of the nerve root. A figure illustrating this grading system can be found elsewhere [7].

Nerve root compromise at the level of the lateral recess in patients with disc herniation was evaluated based on the MR grading system of Pfirrmann et al. [8] The grading system evaluates the relationship between the nerve root and the adjacent intervertebral disc and is reported as follows: Grade 0—no compromise; Grade 1—contact of disc material with the nerve root; Grade 2—contact of disc material and deviation of the nerve root; and finally Grade 3—compression and subsequent deformity of the nerve root. In our study, this grading was used to define the relationship between the nerve root and a possible disc herniation, disc bulging, and an osteoligamentous narrowing of the lateral recess. A figure illustrating this grading system can be found elsewhere [8].

### 2.4. Statistical Analysis

Inter-readout and inter-sequence agreement was quantified with Kappa statistics. A kappa value of less than 0.20 was considered slight; 0.21–0.40, fair; 0.41–0.60, moderate; 0.61–0.80, substantial; and 0.81 or greater, nearly perfect agreement [7]. Furthermore, Wilcoxon signed-rank tests were used to check for differences in the distribution of scores between the sequences. Two-tailed *p*-values < 0.05 were considered statistically significant. All statistical analyses were performed with the R statistical software (version 4.0.2; R Foundation for Statistical Computing, Vienna, Austria, https://www.R-project.org/, accessed date: 20 November 2021).

## 3. Results

**Figure 1:**
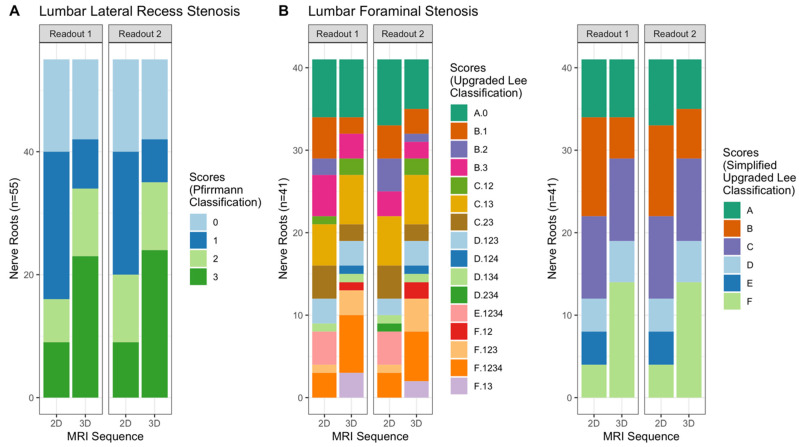
Stacked barplots for the visualization of grading scores stratified by readout and sequence. (**A**) shows the scores for the grading of lumbar lateral recess stenosis and (**B**) shows the grading of lumbar foraminal stenosis. Note the shift toward higher grade stenosis on 3D imaging.

**Figure 2:**
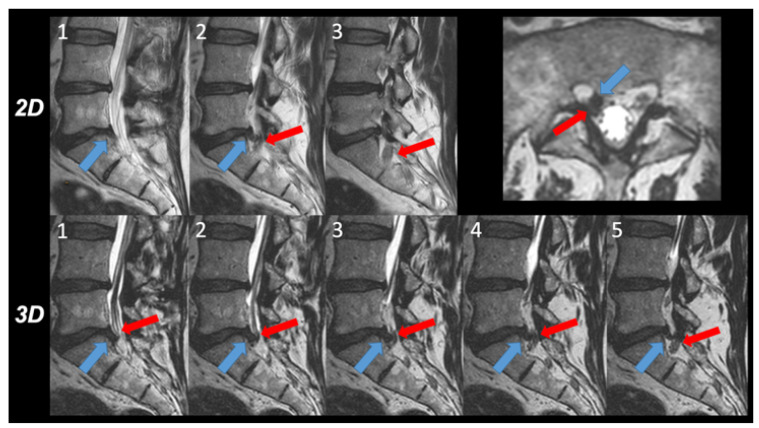
Intra-individual comparison of the 2D and 3D images. The relationship between disc herniation (blue arrow) and nerve root (red arrow) within the lateral recess right side was evaluated on three consecutive 2D T2w TSE images (upper row 1 to 3) and on five 3D T2w TSE images (lower row 1 to 5) depicting each second image. On 2D T2w TSE images, a contact between the disc herniation and the nerve root is depicted in image 2, graded as 1 according to the Pfirrmann grading system. However, in 3D T2w TSE images, a deviation and compression of the nerve root, corresponding to grade 3, was obvious in images 2, 3, and 4 and verified in the transverse thin slice reformatted image.

**Figure 3:**
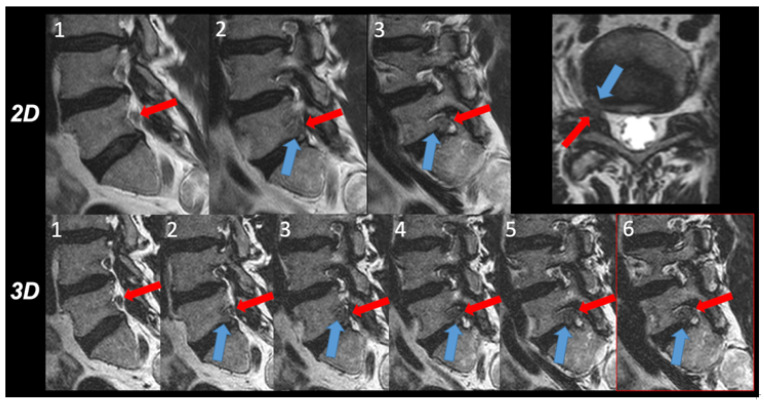
Intra-individual comparison of the 2D and 3D images. The relationship between disc herniation (blue arrow) and nerve root (red arrow) within the intervertebral foramen right side was evaluated in three consecutive 2D T2w TSE images (upper row 1 to 3) and in six 3D T2w TSE images (lower row 1 to 5) depicting each third to fourth image. In 2D T2w TSE images, contact between the disc herniation and the nerve root is depicted in images 2 and 3, however, no nerve root compression was appreciated. In 3D T2w TSE images, however, a compression of the nerve root due to the intraforaminal disc herniation was obvious in images 3 to 6 and verified in the transverse thin slice reformatted image.

A detailed overview of scores is provided in Figure 1 and Table 1. Representative image examples are shown in Figure 2 and Figure 3.

Inter-readout agreement of scores was almost perfect (kappa = 0.935/0.945 for the grading of lateral recess stenosis on 2D and 3D T2w TSE images, respectively, and kappa = 0.823/0.933 for the grading of lumbar foraminal stenosis in the 2D and 3D T2w TSE images, respectively).

There were significant and substantial differences in scores between the 2D and 3D T2w TSE images for both the grading of lateral recess stenosis (*p* < 0.001 for both readouts) and lumbar foraminal stenosis (*p* < 0.001 for both readouts).

Specifically, for the grading of lateral recess stenosis, inter-sequence agreement was moderate with kappa = 0.55/0.577 for readouts 1 and 2, respectively. Importantly, the percentage of high degree stenosis with nerve root deformity (grade 3) increased from 16.4%/16.4% to 41.8%/43.6% from 2D to 3D images for readout 1/readout 2, respectively.

For the grading of lumbar foraminal stenosis, inter-sequence agreement was also moderate with kappa = 0.543/0.572 for readouts 1 and 2, respectively. Importantly, the percentage of high degree stenosis with nerve root deformity (grade F) increased from 9.8% to 34.1% from 2D to 3D T2w TSE images for both readouts.

## 4. Discussion

In this intra-individual comparison study, we compared a high-resolution 3D T2w TSE MRI sequence with a standard-resolution 2D T2w TSE MRI sequence for the grading and visualization of lumbar nerve root compromise in patients with unilateral single radiculopathy. First, our results showed that both grading systems (i.e., 4-point grading system for lumbar lateral recess stenosis and 6-point grading system for lumbar foraminal stenosis) exhibited a high inter-readout agreement for both 2D and 3D T2w TSE imaging. Second, we showed that with high-resolution 3D T2w TSE imaging, higher grade foraminal stenoses and nerve root compression in lateral recesses were diagnosed in a higher number of nerve roots compared with the standard-resolution 2D T2w TSE imaging.

Lower back pain and radiculopathy are considered major public health problems worldwide, causing considerable morbidity and disability [14,15,16]. MRI is widely considered the most important imaging modality to assess degenerative changes of the spine. Importantly, in our experience, spine surgeons increasingly consider the MRI findings aside from the clinical assessment to decide on whether to operate or not on patients with clinically symptomatic radiculopathy. Thus, it is essential to acquire high-quality MR images in order to detect even the smallest degenerative pathologic changes potentially causing and explaining clinical symptomatology.

With the introduction of compressed sensing technology [12], high-resolution imaging has become feasible without compromising scan time efficiency [11]. Specifically, in this study, we implemented a compressed SENSE accelerated near-isotropic 3D T2w TSE sequence in sub-millimeter resolution. Due to the near-isotropic nature of the sequence, transverse and coronary reformations could be computed from a single acquisition. Furthermore and importantly, the nerve roots’ course could be meticulously tracked in much more detail, for example, the lumbar foramen is depicted in 15–20 image slices instead of only 2–3 slices with standard-resolution 2D T2w TSE imaging. For the diagnosis of nerve root compromise, this opens up the possibility of clearly visualizing the nerve root along its entire path in all directions from a single image acquisition.

Our results indicate that with high-resolution 3D imaging, high degree stenoses both in the lateral recess as well as in the intervertebral foramen become apparent in a higher number of nerve roots than with standard-resolution 2D imaging. In this study, we only enrolled patients with unilateral single radiculopathy and clear clinical symptomatology as determined in clinical examination. In such patients, one would expect high-grade stenosis to be present and thus, that an increased diagnosis of high-grade stenosis on high-resolution imaging would correspond to a higher diagnostic accuracy. Nevertheless, it should be emphasized that we did not include surgical correlation as a potential gold-standard index test to definitively ascertain nerve root compromise.

Previous studies have assessed the potential role of (high-resolution) 3D imaging for the diagnosis of spinal pathology and nerve root compromise [17,18,19,20,21]. As an example, Sung et al. compared a high-resolution, isotropic 3D T2w TSE SPACE sequence with a standard 2D T2w sequence for the diagnosis of lumbar nerve root compromise. Interestingly, the authors did not observe a significant advantage of their high-resolution 3D sequence for the diagnosis of nerve root compromise compared to their standard 2D sequence [18].

These findings differ from the results of our study. At least for the diagnosis of foraminal stenosis, the choice of grading system may explain certain discrepancies. Sung et al. used a 4-point grading system similar to the one described by Lee et al. to quantify the degree of change within the lumbar intervertebral foramen. In contrast, we used an updated 6-point grading system designed for high-resolution imaging that enables a comprehensive description of even the smallest changes in the lumbar intervertebral foramen. Consequently, our 6-point grading system may have been more sensitive in quantifying and describing even small changes in the foramen, thus enabling a more thorough comparison between 2D and high-resolution 3D T2w TSE images. In this regard, it should be noted that the inter-readout agreement of our updated 6-point grading system was excellent for both the 2D and 3D T2w TSE images, thus confirming and even extending on the findings of previous studies, as to date, the reproducibility of this grading system has only been confirmed for high-resolution 3D imaging.

Furthermore, potential differences of the pulse sequences should be addressed. Specifically, the SPACE sequence from Sung et al. [18] and our 3D sequence are technically comparable. Both sequences were based on the principles of long echo trains combined with variable flip angle sweeps and acceleration with either GRAPPA, or compressed SENSE [10,11,13,22] in our case [23]. However, our sequence was 2 min shorter. Potentially, this was only possible due to compressed SENSE, since this technique allows for a much higher acceleration than conventional parallel imaging such as GRAPPA or SENSE. To the best of our knowledge, this is the first paper to use a compressed SENSE accelerated sequence for the diagnosis of nerve root compromise. Thus, besides differences in the study cohort, the differing pulse sequence parameters, the occurrence of motion artifacts and potentially, differences in image resolution may have contributed to discrepancies in the results.

Our study has the following limitations: First, we only included a limited number of subjects. Second, surgical correlation and standardized assessment of radiculopathy was not available, thus prohibiting us from quantitatively correlating imaging grading scores with various degrees of clinical symptomatology. The reasons for this were that a considerable number of patients underwent further diagnostic workup or surgical intervention at external centers, thus prohibiting us from keeping track of the further disease course or outcome. Third, we acknowledge certain limitations of our statistical analysis. Specifically, we used Kappa statistics to quantify the agreement of scores. We are aware that different statistical approaches may render other results. Fourth, due to the high number of lumbar MRI examinations at our institutions, we only included patients with lumbar nerve root compromise. However, the topic of this study may also be relevant for patients with nerve root compromise of the thoracic and cervical spine. Future studies should address this. Fifth, it should be mentioned that there may also be drawbacks to using high-resolution 3D imaging that were not considered for this study. Specifically, image interpretation of high-resolution 3D images may be considerably prolonged compared to standard-resolution 2D images. Furthermore, we did not assess the true resolution of the 3D sequence. A dedicated phantom experiment could provide accurate data on this matter, but we considered this beyond the scope of this paper. Sixth, it should be mentioned that our high-resolution 3D sequence requires a high-performance MRI system, which restricts its general availability to some extent. Seventh, we did not assess the image quality of the 2D and 3D sequences. However, previous studies have been published on this matter, corroborating the fact that the 3D sequence provides good and diagnostic image quality [9]. Eighth, we did not perform a quantitative evaluation of nerve root compromise. We are, however, aware that quantitative evaluation methods may represent a promising method of further improving the diagnostic yield of a radiological examination [24,25,26]. Finally, patients were enrolled due to their clinical symptomatology after a comprehensive clinical examination. Despite these measures, it may be possible that patients with inflammatory radiculitis were included, which would have introduced a bias to the study.

## 5. Conclusions

In conclusion, we first showed that an updated 6-point grading system for lumbar foraminal stenosis is reproducible and reliable both for high-resolution 3D and standard-resolution 2D T2w TSE imaging. Future studies should assess the correlation between the grading scores of this updated 6-point grading system and scores from a standardized clinical assessment of radiculopathy. Second, we provide evidence that high-resolution 3D imaging outperforms standard-resolution 2D T2w TSE imaging for the visualization of lumbar nerve root compromise.

## Figures and Tables

**Table 1:** Overview of grading scores. For the sake of clarity, only the simplified grading scores from lumbar foraminal stenosis are shown.

	Readout 1		Readout 2	
	2D T2w TSE	3D T2w TSE	2D T2w TSE	3D T2w TSE
Lumbar Lateral Recess Stenosis (n = 55)	Grade 0: 15 (27.3%)	Grade 0: 13 (23.6%)	Grade 0: 15 (27.3%)	Grade 0: 13 (23.6%)
	Grade 1: 24 (43.6%)	Grade 1: 8 (14.5%)	Grade 1: 20 (36.4%)	Grade 1: 7 (12.7%)
	Grade 2: 7 (12.7%)	Grade 2: 11 (20%)	Grade 2: 11 (20%)	Grade 2: 11 (20%)
	Grade 3: 9 (16.4%)	Grade 3: 23 (41.8%)	Grade 3: 9 (16.4%)	Grade 3: 24 (43.6%)
Lumbar Foraminal Stenosis (n = 41)	Grade A: 7 (17.1%)	Grade A: 7 (17.1%)	Grade A: 8 (19.5%)	Grade A: 6 (14.6%)
	Grade B: 12 (29.3%)	Grade B: 5 (12.2%)	Grade B: 11 (26.8%)	Grade B: 6 (14.6%)
	Grade C: 10 (24.4%)	Grade C: 10 (24.4%)	Grade C: 10 (24.4%)	Grade C: 10 (24.4%)
	Grade D: 4 (9.8%)	Grade D: 5 (12.2%)	Grade D: 4 (9.8%)	Grade D: 5 (12.2%)
	Grade E: 4 (9.8%)	Grade E: 0 (0%)	Grade E: 4 (9.8%)	Grade E: 0 (0%)
	Grade F: 4 (9.8%)	Grade F: 14 (34.1%)	Grade F: 4 (9.8%)	Grade F: 14 (34.1%)

## Data Availability

Data are available from the corresponding author upon reasonable request.
